# Valganciclovir as Add-On to Standard Therapy in Secondary Glioblastoma

**DOI:** 10.3390/microorganisms8101471

**Published:** 2020-09-24

**Authors:** Giuseppe Stragliotto, Mattia Russel Pantalone, Afsar Rahbar, Cecilia Söderberg-Nauclér

**Affiliations:** 1 Department of Medicine, Solna, Microbial Pathogenesis Unit, Karolinska Institutet, 17164 Stockholm, Sweden; giuseppe.stragliotto@sll.se (G.S.); afsar.rahbar@ki.se (A.R.); 2 Division of Neurology, Karolinska University Hospital, 17177 Stockholm, Sweden

**Keywords:** glioblastoma, secondary glioblastoma, valganciclovir, cytomegalovirus

## Abstract

Patients with glioblastoma have a very poor prognosis despite aggressive therapeutic strategies. Cytomegalovirus has been detected in >90% of glioblastoma tumors. This virus can affect tumor progression and may represent a novel glioblastoma therapy target. We report, here, a retrospective survival analysis of patients with secondary glioblastoma who were treated with the anti-viral drug valganciclovir at Karolinska University Hospital in Stockholm. We performed survival analyses of eight patients with secondary glioblastoma who were treated with a standard dose of valganciclovir as an add-on to second-line therapy after their disease progression to glioblastoma. Thirty-six patients with secondary glioblastoma admitted during the same time period who received similar treatment and care served as contemporary controls. The patients treated with valganciclovir showed an increased median overall survival after progression to glioblastoma compared with controls (19.1 versus 12.7 months, *p* = 0.0072). This result indicates a potential positive effect of valganciclovir in secondary glioblastoma, which is in agreement with our previous observation that valganciclovir treatment improves the outcomes of patients with newly diagnosed glioblastoma. Larger randomized studies are warranted to prove this hypothesis.

## 1. Introduction

Glioblastoma is the most frequent and deadliest form of brain cancer. Primary glioblastoma comprises about 90% of cases and originates de novo, while secondary glioblastomas (10%) are considered to arise from less malignant tumor precursors such as a low-grade diffuse astrocytoma or oligodendrioglioma that progresses into a high-grade lesion. Primary and secondary glioblastomas are almost impossible to differentiate by histopathology, but they have different genetic and epigenetic profiles, wherefore they are considered as different disease entities [1].

Primary glioblastoma primarily occurs in older patients (mean age, 62–64 years) and typically shows epidermal growth factor receptor (EGFR) overexpression, PTEN mutations, cyclin-dependent kinase inhibitor (CDKN2A) (p16) deletion, and, sometimes, mouse double minute 2 homolog (MDM2) amplification [2]. Secondary glioblastoma patients are generally younger (mean age, 45–48 years), and their tumors are characterized by TP53 mutations, 1p19q loss and isocitrate dehydrogenase (IDH) mutations, the latter suggested to be present in 70–80% of cases [3]. Patients with IDH mutations are considered to have a better prognosis [3], demonstrating an about twice-as-long median OS as patients with primary glioblastoma [4,5,6]. Among 14 patients with IDH1 or IDH2 mutations, the median OS was reported to be 31 months, as compared to 15 months in 115 patients with IDH1 wild-type primary glioblastoma tumors [4]. Another study found similar higher survival rates in 36 patients with IDH1-mutant tumors—27.1, versus 11.3 months in 371 patients with IDH1 wild-type tumors [5]. Other studies not defining IDH mutation status did not show a higher survival rate for secondary glioblastoma patients as compared to those with primary disease (7.8 or 11 months, respectively) [2,7]. There is, however, remarkably sparse literature on the outcomes of patients with secondary glioblastoma; most studies have focused on patients with primary glioblastoma or low-grade tumors. Although no defined standard treatment exists for these patients, therapeutic interventions seem to be beneficial also in patients with secondary glioblastoma, and patients receiving complete tumor resection and adjuvant radio-chemotherapy seem to survive longer [7].

The etiology of primary or secondary glioblastoma is unknown. Human cytomegalovirus (CMV) is a common beta herpes virus that rarely causes symptomatic disease in healthy individuals but that has been detected in several solid tumors, including gliomas and glioblastomas [8,9]. Although not considered oncogenic, this virus can cause all the ten hallmarks of cancer, and certain CMV strains have been shown to induce cellular transformation in vitro and in vivo [10]. Patients with glioblastoma who have lower loads of CMV in their tumor at diagnosis appear to survive longer [11,12], and anti-CMV therapy in mouse models decreased tumor growth and improved survival [13]. CMV may, therefore, represent a target of therapy in glioblastoma. In support of this statement, we recently reported that 102 patients with newly diagnosed primary glioblastoma treated with the anti-viral drug valganciclovir had a significantly improved OS as compared with 231 contemporary control patients with similar characteristics who were treated at the same institution (24.1 vs. 13.3 months, *p* < 0.0001) [14]. Thus, this virus could represent a treatment target in glioblastoma patients. In the present study, we present a retrospective analysis of the treatment results for valganciclovir as an add-on to second-line treatment in eight patients with secondary glioblastoma, who had significantly longer median survival after progression than 36 control patients receiving similar therapy at our institution.

## 2. Materials and Methods

Eight patients with secondary glioblastoma were treated with valganciclovir as an add-on to second-line therapy between 12 December 2006, and 10 July 2020, at Karolinska University Hospital. Valganciclovir was given at the standard recommended dose: 900 mg twice daily for 3 weeks followed by 900 mg daily. No side effects were associated with valganciclovir treatment. Thirty-six patients with secondary glioblastoma treated at our institution served as contemporary controls; these patients were retrieved by a manual search of patient’s charts. None of them had participated in another clinical trial. While patients may have been missed during the manual search, we did not perform any active selection of patients. Patient data were anonymized and organized in a database. The study was conducted in accordance with the Declaration of Helsinki and approved by the regional ethics committee in Stockholm (Dnr: 2016/1426/31/1). We were not required to obtain informed consent from the living patients or relatives of deceased patients for this study. The patient data are in a data file that can be connected to the patient with a code key. The authors followed the treated patients throughout the study.

The Karnofsky performance score (KPS) was assessed in routine clinical examinations. O6-methylguanine-DNA methyltransferase (MGMT) promoter status and p53 and IDH mutations were tested in the pathology department at our hospital and reported when available.

A diagnosis of secondary glioblastoma was made when a pre-existent low-grade glial tumor (astrocytoma or oligodendroglioma grade II) progressed into a high-grade glioblastoma. The progression was assessed radiologically by MRI and pathologically in re-operated patients. The criteria considered to evaluate progression to secondary glioblastoma included the evolution of foci or appearance of contrast enhancement, tumor growth, edema, necrosis and hemorrhage. The other variables considered were age, sex, tumor location, KPS, MGMT status, and p53 and IDH mutation status.

The treatment of patients with low-grade glioma at our institution consists of surgical extirpation when possible followed by radiotherapy (maximum of 60 Gy). In some cases, chemotherapy with lomustine was prescribed. Patients with tumor progression to a high-grade lesion were also, when possible, re-operated and re-irradiated. Second-line chemotherapy mainly included temozolomide, lomustine and/or bevacizumab. Another option was gamma-knife treatment.

The primary endpoint was median overall survival (OS) time after progression to secondary glioblastoma. We also estimated the time to tumor progression (TTP) to secondary glioblastoma and median OS after the first tumor diagnosis. Survival data are presented as Kaplan–Meier estimates calculated form the time of diagnosis. All statistical hypotheses were two-sided, with a significance level of 5%. Significance was determined with the log-rank test; *p* < 0.05 was considered statistically significant. GraphPad Prism (version 8.3) was used for statistical analyses.

## 3. Results

Patients’ characteristics are summarized in Table 1. Among the eight valganciclovir-treated patients, three were men (37.5%), and the median age at first diagnosis was 33.5 (ranging between 27 and 63) years. All of them had astrocytoma grade II as their first diagnosis. First-line treatment included surgery for all eight patients, followed by radiotherapy in seven patients, lomustine in six patients and temozolomide in one patient. The median time to development of glioblastoma was 68.7 months (13.2–131.4 months). The median age at diagnosis of secondary glioblastoma was 43.5 years (range, 30–66 years). The median KPS at progression to glioblastoma was 80. Second-line treatment was heterogeneous: four patients were re-operated, two underwent gamma-knife treatment and six received re-irradiation. Second-line chemotherapy was temozolomide for six patients, two received lomustine, and three were treated with bevacizumab. The IDH mutational status of secondary GBM was known only for two patients, and one was positive. The MGMT promoter status was known for one patient, who had an unmethylated MGMT promoter. The TP53 mutation status had been investigated in five cases in the primary tumors, and four of them had tumors with mutated TP53.

Among the controls, 23 of 36 (63.9%) were men and the median age at first diagnosis was 38 years (21–76 years). Thirty-one patients had astrocytoma grade II, and five had oligodendroglioma grade II as the first diagnosis. First-line treatment included surgery for all patients, followed by radiotherapy in 27 patients and lomustine in 24 patients. The median time to tumor progression (TTP) to glioblastoma was 62.4 months (4.1–255.1 months). The median age at diagnosis of secondary glioblastoma was 45 years (27–78 years). The median KPS at progression was 80. Second-line treatment was heterogeneous: nine patients were re-operated, seven underwent gamma-knife treatment and 26 received re-irradiation. Second-line chemotherapy was temozolomide in 28 patients and lomustine in nine patients. Bevacizumab was administered to 10 patients in combination with temozolomide or lomustine. The IDH mutation status was known for 11 control patients with secondary glioblastoma, and nine were positive. The MGMT promoter status was known for eight patients, of which three had secondary GBM with a methylated MGMT promoter. The TP53 mutational status was investigated in the primary tumors of 19 patients, of which 15 had mutated TP53.

**Figure 1:**
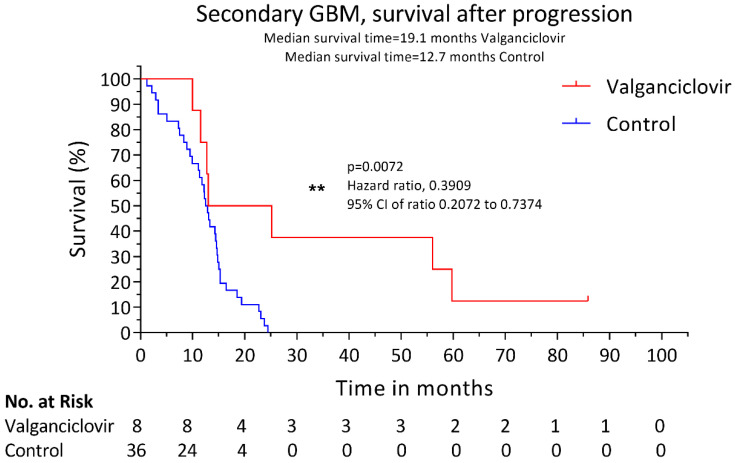
Kaplan–Meier estimates of median overall survival (OS) after progression. Estimated median OS after progression to glioblastoma for 8 patients with secondary glioblastoma who received valganciclovir therapy (red) and for 36 contemporary controls who received similar second-line therapy (blue). **: the level of significance of the *p* value (*p* = 0.0072) indicated with the symbol ** by the statistical program used, Graphpad v. 8.

**Figure 2:**
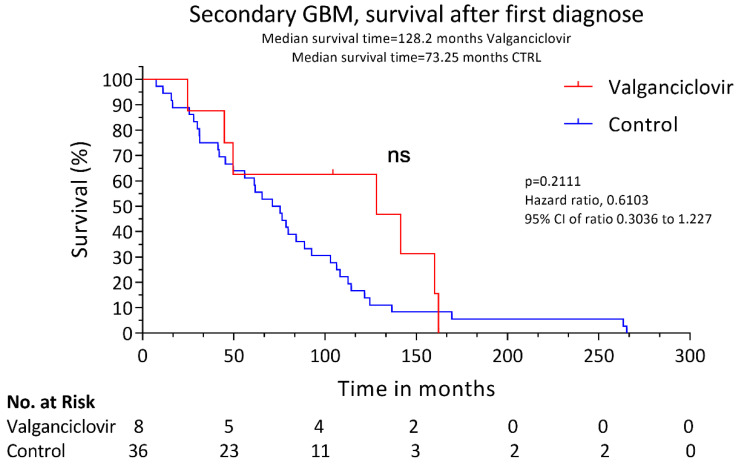
Kaplan–Meier estimates of overall survival after first tumor diagnosis. Estimated median OS survival after first tumor diagnosis for 8 patients with low-grade glioma that progressed later into secondary glioblastoma who received valganciclovir therapy (red) and for 36 contemporary controls who received similar therapy (blue).

The patients were treated with valganciclovir after glioblastoma diagnosis at the standard dose of 900 mg twice daily for 3 weeks followed by 900 mg daily. The drug was well tolerated, and no relevant side effects were observed. The median survival after progression to glioblastoma was longer in valganciclovir-treated patients than in controls (19.1 versus 12.7 months, *p* = 0.0072) (Figure 1). The two-year survival rate after progression to glioblastoma was also higher in patients receiving valganciclovir (37.5% vs. 2.8%, *p* = 0.0203). The time to progression to glioblastoma was similar in control patients and in those who had subsequently been treated with valganciclovir (62.4 versus 68.7 months, respectively, *p* = 0.7775). The OS was 128.2 months in valganciclovir-treated patients and 73.25 months in controls, but the difference was not significant; *p* = 0.2111 (Figure 2). The patients who underwent a re-operation after the diagnosis of secondary GBM showed no improvement in survival compared to non-re-operated patients in the valganciclovir group (*p* = 0.9659) or in the controls (*p* = 0.1540).

## 4. Discussion

Since 2006, we have treated 139 patients with glioblastoma with valganciclovir as an add-on to standard therapy; this drug treatment aims to target the negative effects of CMV in glioblastoma, an infection that is present in the majority of these tumors. We recently reported a retrospective data analysis demonstrating enhanced survival among 102 patients with primary glioblastoma who received valganciclovir as an add-on to standard therapy compared with 231 control patients [14]. Here, we also report that eight patients with secondary glioblastoma who were treated with valganciclovir as an add-on to second-line therapy survived significantly longer than controls after their progression to glioblastoma.

To our knowledge, this is the first report describing the use of valganciclovir in patients with secondary glioblastoma. In accordance with the survival data for patients with newly diagnosed glioblastoma, who survived longer when treated with valganciclovir than patients receiving only the standard of care, we also show that patients with secondary glioblastoma may benefit from anti-viral therapy. These patients survived 6.4 months longer than their controls (*p* = 0.0072). As was reported by Hamisch et al., we did not observe a better prognosis among patients with secondary glioblastoma than among patients diagnosed with primary glioblastoma. However, we did not have information about IDH mutation status for most patients, which has been shown to influence the survival chances for these patients [3,4,5,6]. Although this small patient cohort only included eight valganciclovir-treated patients, their benefit from this drug was statistically significant. Thus, targeting CMV in glioblastoma continues to show potential treatment benefits, representing a promising new therapy option, in need of further evaluation in randomized trials.

Can CMV promote tumor progression and thereby explain the potential positive effect of anti-viral therapy in glioblastoma patients? CMV is present in low-grade gliomas, but to a lesser extent than in glioblastomas [9]. The level of CMV appears to be linked to tumor aggressiveness, and the virus is more abundantly found in high-grade gliomas/glioblastomas [12,15,16]. CMV may, therefore, promote progression from low-grade to high-grade gliomas. In experimental models, CMV proteins can cause all the ten hallmarks of cancer, and certain viral strains have been shown to induce oncogenic transformation [17]. For example, virus-infected cells exhibit chromosomal instability and cellular stress, with an impaired DNA damage response and reduced DNA repair machinery, which can result in gene mutations [18,19]. This virus also regulates p53, Rb, cyclins and p21 functions that can impact cell cycle control [10,20,21]. The further reprogramming of cellular metabolism according to the Warburg effect and viral activation of AKT, MAPK, PI3K and mTOR will promote protein translation and the production of biomass and energy, which allows for a high rate of cell division [22,23]. CMV also controls the production of growth factors such as PDGF, IGF, EGFR and VEGF and several of their receptors along with virus-encoded GPCRs such as US28, which promote cellular proliferation, migration and angiogenesis [10,24]. CMV further regulates host cell gene expression through epigenetic regulation, and the viral IE proteins act as transcription factors to control host gene expression, as exemplified by the binding of IE72 to the hTERT promotor and induction of telomerase activity to elongate telomeres and prolong cell survival [25]. The regulation of hypoxic signaling mechanisms further links this virus to cancer promotion [26,27]. CMV-induced inflammation, via the induced production of pro-inflammatory cytokines and expression of COX2 and 5-LO, can also enhance tumor aggressiveness [28,29,30,31], while simultaneously providing numerous immune evasion mechanisms to counteract the innate and adaptive immune responses to protect tumor cells from elimination by the immune system [32]. At least five CMV-encoded proteins and the long non-coding RNA β2.7 are anti-apoptotic and further promote cell survival [33]. Thus, the presence of this virus in glioblastoma may confer higher aggressive potential to virus-positive tumor cells.

However, although all these mechanisms would promote cancer development and progression, CMV is not included among the group of classic oncogenic viruses. The reason is mainly due to the lack of robust evidence that CMV induces the oncogenic transformation of normal cells. In the mid-1970s, Rapps’ group demonstrated the oncogenic transformation of normal fibroblast cells and tumor establishment in immunodeficient mice [34]. More recently, Herbein and colleagues isolated a slowly growing CMV strain HCMV-DB from a pregnant woman that induced the transformation of normal mammary epithelial cells in vitro. Implantation into mice gave rise to triple-negative breast tumors [35,36]. In another study, the expression of the viral chemokine homologue US28 in NIH-3T3 cells induced tumor formation, and transgenic mice expressing US28 in the bowel developed tumors when challenged with an inflammatory stimulus [37]. When US28 was expressed in glioblastoma cells, tumors were more rapidly formed, a phenomenon that could be prevented by treatment with US28-specific nanobodies [38,39]. In another mouse model, CMV promoted glioblastoma development, which was prevented with anti-viral therapy [13]. Tumor growth was also decreased in CMV-positive medulloblastoma and neuroblastoma xenografts by valganciclovir treatment without or in combination with a COX-2 inhibitor [28]. These basic scientific and animal model data provide the rationale for the use of anti-CMV therapy to prevent tumor progression in glioblastoma patients. If the viral mechanisms that promote more-aggressive tumor phenotypes and the establishment of new tumor clones could be halted by anti-viral therapy, the prognosis and survival of patients diagnosed with CMV-positive tumors may improve.

We were the first to treat glioblastoma patients with anti-CMV therapy. In a randomized, small, hypothesis-generating, double-blinded, phase I/II clinical trial, initiated in 2006, we observed trends toward smaller tumor growth in valganciclovir-treated patients [40]. The study was too small to provide statistically significant results (*n* = 42, of which 22 received valganciclovir therapy). However, in exploratory analyses, we observed that patients who were treated with valganciclovir had a longer median OS than patients with only standard-of-care treatment (24.1 versus 13.1 months, *p* < 0.0001). In 2013, we reported, in a letter to the New England Journal of Medicine, data from the cohort then extended to 50 valganciclovir-treated patients with glioblastoma [41]. This analysis also demonstrated significantly improved survival among valganciclovir-treated patients. While awaiting a possibility to find financial support for a randomized trial, the number of valganciclovir-treated patients increased to 139 patients by 2019. Among these, 102 had primary glioblastoma, and the median OS rate was very similar to that in the first trial: 24.1, versus 13.3 months in 231 contemporary control patients (*p* < 0.0001) [14]. Both patients with methylated MGMT and those with an unmethylated MGMT promoter showed potential benefit from valganciclovir treatment. It is very unusual for patients diagnosed with unmethylated MGMT tumors to respond to any therapy given to them, which implies that anti-CMV therapy likely affects their tumor cells. Mitchell and Sampsons’ teams have also shown promising effects from dendritic cell therapy using CMV-pp65-mRNA-pulsed dendritic cells in patients with glioblastoma, which provides further support for the hypothesis that CMV can represent a target for therapy in patients with glioblastoma [42,43]. In contrast to the promising effects of valganciclovir as a treatment to improve the survival of patients with glioblastoma, the chances of surviving on other therapies has remained fairly unchanged for patients with glioblastoma since 2005 [44]. Evaluated treatments with bevacizumab [45], dendritic cell vaccination [46], EGFR-targeted immunotherapy [47], and integrin or mTOR inhibitors [48] have, to date, all failed to show any benefit for patients with glioblastoma, wherefore patients have remained on the same standard of therapy with surgery and radiochemotherapy with temozolomide. Only tumor-treating fields show some positive effects on survival, and this therapy is now emerging as a standard therapy in many countries [49]. Therefore, the promising effects of anti-CMV treatment strategies need to be evaluated in randomized trials.

Such clinical studies should also seek to elucidate the mechanisms by which valganciclovir prevents tumor growth and if all patients respond equally to this therapy. Although the most plausible anti-tumor effect is believed to be mediated by the action of valganciclovir on virus replication, this does not exclude other mechanisms of action due to the possible off-target effects of this medication. Valganciclovir is a pro-drug to ganciclovir, which is activated in vivo and particularly in CMV-infected cells by the kinase UL97, which produces the tri-phosphate active form of ganciclovir, which acts as a nucleoside analogue that is incorporated into the DNA, halts DNA replication and induces apoptosis [50,51]. Under such circumstances, tumor cell division may also be prevented. However, in an animal model, we observed that the tumor growth of human xenografted tumors was only prevented in CMV-positive tumors but was not evident in a CMV-negative tumor [28]. This suggests that valganciclovir´s main mechanism of inhibiting tumor growth is mediated via the prevention of virally induced mechanisms. Such mechanisms are considered to be similar for any CMV-positive tumor, wherefore valganciclovir may prevent the tumor growth of other CMV-positive tumors including medulloblastoma, neuroblastoma and breast, colon, prostate and ovarian cancers as well, as these are shown to be highly positive for CMV. Of note, cidofovir, a nucleotide analogue with a mechanism of action similar to that of ganciclovir and used to treat serious CMV infections, has been shown to augment radiation-induced DNA damage and to exert anti-neoplastic effects in glioblastoma, even in the absence of CMV [52]. Further studies are warranted to investigate a possible interaction between radiotherapy and valganciclovir in patients with cancer. As in any retrospective study, the patient selection criteria could affect the treatment results, especially in a limited cohort as we report here. The first- and second-line treatments were also heterogeneous and may have impacted the prognosis. Another important limitation is the relatively small number of patients in both the treatment and control groups and the lack of knowledge of their IDH mutation status. Nevertheless, similarly to the larger number of patients with primary glioblastoma, valganciclovir-treated patients with secondary glioblastoma survived significantly longer than their controls. Thus, valganciclovir may be a promising new therapeutic option for patients with secondary glioblastoma. We are currently conducting a randomized double-blind clinical trial to assess the effect of valganciclovir in 220 patients with primary glioblastoma (VIGAS2, Clinical Trials.gov Identifier: NCT04116411). Our observations suggest that a randomized study would also be warranted for patients with secondary glioblastoma to evaluate this promising treatment option further.

## 5. Conclusions

Our study shows that valganciclovir may improve survival in patients with secondary glioblastoma. The data are consistent with reports on the valganciclovir treatment of patients with primary glioblastoma and offer the first evidence of a possible beneficial effect of valganciclovir therapy in secondary glioblastoma, an observation that necessitates a larger, randomized confirmatory trial.

## Figures and Tables

**Table 1:** Demographic and clinical characteristics.

	Secondary Glioblastoma	
Characteristics	Controls ^1^ *n* = 36 (%)	Valganciclovir *n* = 8 (%)
**Age at LGG, years**		
Median	38	33.5
Range	21–76	27–63
**Age at GBM, years**		
Median	45	43.5
Range	27–78	30–66
**Sex**		
Women	13 (26.1)	5 (62.5)
Men	23 (63.9)	3 (37.5)
**Race** Caucasian		
	(100)	(100)
**TP53 status**		
Wild type	4 (11.1)	1 (12.5)
Mutated	15 (41.7)	5 (62.5)
NA	17 (47.2)	2 (25)
**Tumor location**		
Temporal	11 (30.5)	2 (25)
Frontal	14 (38.9)	4 (50)
Parietal	9 (25.0)	2 (25)
Occipital	1 (2.8)	0
Other	0	0
**Primary treatment**		
Surgery		
Radical resection	13 (36.1)	2 (25)
Partial resection or biopsy	23 (63.9)	6 (75)
Radiotherapy	30 (83.3)	7 (87.5)
Lomustine	24 (55.6)	6 (75)
Temozolomide	3 (8.3)	1 (12.5)
**Second-line therapy**		
Re-operation	9 (25)	4 (50)
Not re-operated	27 (75)	4 (50)
Gamma-knife treatment	7 (19.4)	2 (25)
Re-irradiation	26 (72.2)	6 (75)
Lomustine	9 (25)	2 (25)
Temozolomide	28 (77.8)	6 (75)
Bevacizumab	10 (27.8)	3 (37.5)

^1^ Controls received standard-of-care treatment; LGG, low-grade gliomas; GBM, glioblastoma; NA, not known; *n*, number.
